# Access to percutaneous transluminal coronary angioplasty and 30-day mortality in patients with incident STEMI: Differentials by educational level and gender over 11 years

**DOI:** 10.1371/journal.pone.0175038

**Published:** 2017-04-06

**Authors:** Laura Cacciani, Nera Agabiti, Anna Maria Bargagli, Marina Davoli

**Affiliations:** Department of Epidemiology, Lazio Regional Health Service, Rome, Italy; Universitatsklinikum Freiburg, GERMANY

## Abstract

**Background:**

Socioeconomic status and gender are associated with access to cardiac procedures and mortality after AMI, also in countries with universal health care systems. Our objective was to evaluate the association and trends of educational level or gender and the following outcomes: 1) access to PTCA; 2) 30-day mortality.

**Methods:**

We conducted an observational study based on 14,013 subjects aged 35–74 years, residing in Rome in 2001, and hospitalised for incident STEMI within 2012 in the Lazio region. We estimated adjusted ORs of educational level or gender and: 1) PTCA within 2 days after hospitalisation, 2) 30-day mortality. We evaluated time trends of outcomes, and time trends of educational or gender differentials estimating ORs stratified by time period (two time periods between 2001 and 2012). We performed a hierarchical analysis to account for clustering of hospitals.

**Results:**

Access to PTCA among patients with incident STEMI increased during the study period, while 30-day mortality was stable. We observed educational differentials in PTCA procedure only in the first time period, and gender differentials in both periods. Patterns for 30-day mortality were less marked, with educational differentials emerging only in the second period, and gender differentials only in the first one, with patients with low educational level and females being disadvantaged.

**Conclusions:**

Educational differentials in the access to PTCA disappeared in Lazio region over time, coherently with scientific literature, while gender differentials seem to persist. It may be important to assess the role of female gender in patients with STEMI, both from a social and a clinical point of view.

## Introduction

Although ischemic heart diseases are a leading cause of morbidity and mortality in most European countries [1] and worldwide [2], decreasing patterns of incidence and mortality in acute myocardial infarction (AMI) have been observed over time [3]. This could be due in part to the reduction of known cardiovascular risk factors [4], and to a decrease in the incidence of ST-segment elevation myocardial infarction (STEMI) [5], which exposes patients to a higher risk of short-term mortality compared to non–ST-segment elevation myocardial infarction (NSTEMI). On the other hand, a nationwide observational study conducted in 2007 in France showed that patients with NSTEMI and STEMI have comparable in-hospital and long-term prognoses [6].

The increased uptake of evidence-based treatments, including timely use of revascularization procedures such as percutaneous coronary interventions (PCI) in STEMI, may also have contributed to the decreasing mortality trend [7].

Unfortunately, there is evidence showing that improvements in cardiovascular health are not equally distributed across populations, and that low socioeconomic status or educational status negatively affect either access to cardiac procedures [8] or short- [9] and long-term mortality after AMI [10, 11], even in countries with universal health care systems [12, 13]. Also gender differentials in treatments or mortality have been reported in Europe [9, 14] and in the US [15, 16].

In Italy, improvements in the short-term prognosis of patients hospitalised for AMI have been observed in 2001–2011 [17]. However, only few studies investigated socio-demographic differentials in access to therapeutic procedures and outcomes after AMI, with findings changing over time. A study conducted in Rome in 1998–2000 on patients with first acute coronary event showed limited access of disadvantaged patients to recanalization procedures; instead, it found weak association between deprivation status or educational level and short term fatality, and no association with one-year prognosis [18]. A study conducted in Tuscany in 2001–2008 showed a reduction of socioeconomic differentials in percutaneous transluminal coronary angioplasty (PTCA) utilisation among patients with STEMI [19]. Another study conducted in Piedmont in 2008 on patients with AMI, despite gender and socioeconomic differences in the use of revascularization, did not find gender or social disparities in either short- or long-term mortality, also stratifying by STEMI and NSTEMI [20]. A more recent population-based study in Tuscany found differences in coronary reperfusion therapy of hospitalised patients with AMI by gender, showing that women were less treated than men [21].

It remains unclear whether there are socioeconomic and gender differentials in access to effective coronary interventions and post-AMI mortality, and if such differences are due to unequal treatment. Updated data on temporal trends in the use of cardiac procedures and in mortality on large scale, which may help to disentangle this question, are very limited.

The objective of this study was to evaluate temporal trends of the association of educational level attained and gender with access to PTCA or 30-day mortality. We used data from the Rome Longitudinal Study (RoLS) [22], a cohort of residents in Rome in 2001 followed up until 2012, taking into account comorbidities and other relevant risk factors. We chose the PTCA procedure because it is the treatment of choice for STEMI patients [23, 24], and short-term mortality because it is considered a valid and reproducible measure related to appropriateness and effectiveness of diagnosis and treatment of AMI [25].

## Material and methods

### Ethics statement

The RoLS is part of the National Statistical Program for the years 2011–2013 and was approved by the Italian Data Protection Authority. It was not necessary to obtain patients’ consent because we used data already collected at the beginning of the study and the data were analysed anonymously. Individuals cannot be identified either directly or through identifiers, and results are shown in aggregate form.

### Study design and data sources

We conducted an observational study of subjects participating in the RoLS cohort, which includes people residing in Rome on 21 October 2001 (about 2.5 million inhabitants at the 2001 Italian census, National Institute of Statistics). Through record-linkage procedures, conducted under strict control to protect individual privacy, information on socioeconomic characteristics reported in the 2001 Census of the Italian population were collected for 2,118,670 individuals. Vital status was followed up until 31 December 2012 using data of the Rome Municipal Register. During the study period, 214,213 individuals (10.1%) deceased; information on death causes were retrieved from the Regional Mortality Registry for 213,341 (99.6%) individuals, while 284,344 were lost to follow-up due to emigration (13.4%).

### The study cohort

**Fig 1:**
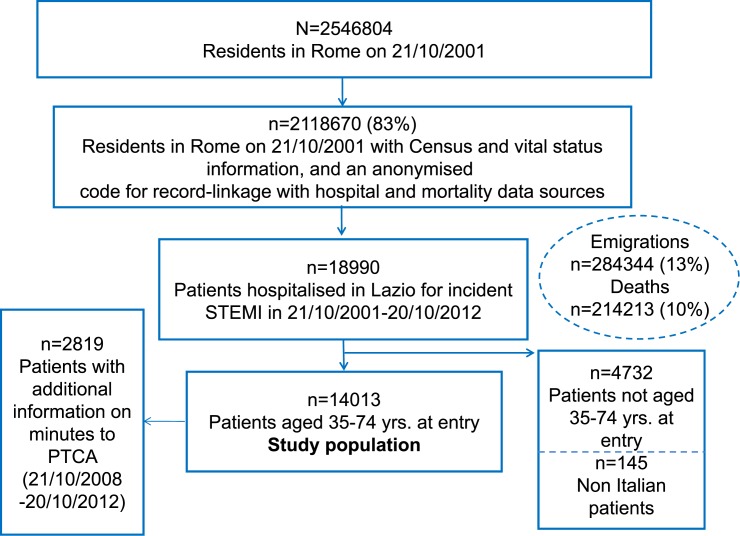
Flow-chart for the selection of the study cohort.

Using the Regional Hospital Discharge Information System, we retrospectively identified and extracted from the RoLs cohort individuals admitted to any hospital in the Lazio region for an incident STEMI (ICD9CM codes 410.x except x = 7,9 in principal or secondary diagnoses) between 21 October 2001 and 20 October 2012. We considered hospital discharge records registered during an additional period of five years before the enrolment date to check if STEMI cases selected were incident. We included only people aged 35–74 years at entry in the RoLS. We excluded foreign citizens, which may have a different chance to be followed up, especially at older ages, obtaining a final study population of 14,013 individuals. Additional information on the time span between hospital admission and PTCA procedure were available from 21 October 2008 to 20 October 2012 in the RAD-esito archive, an extension of the hospital discharge registry. Fig 1 shows the flow-chart for the selection of the study cohort.

### Exposures, risk factors and outcomes

We considered two main exposures: educational level attained (one of the most important dimensions of socioeconomic position) at cohort entry (21 October 2001), classified according to the International Standard Classification of Education (ISCED): no education/primary level (0/1, reference category), lower secondary (2), upper secondary (3), post-secondary or more (4, 5, 6); and gender (reference: females).

In order to evaluate temporal trends, time was analysed according to years or equal time periods.

Demographic information, such as age at event and birthplace (born in Rome vs born elsewhere), and hospitalisations in the two years before the first occurrence of STEMI (for heart, cerebrovascular, liver/chronic digestive, or chronic renal diseases, anemia and coagulation disorders, cancer, COPD—see Table 1 for ICD9CM descriptions and codes) were considered as additional risk factors. In addition, in a hierarchical analysis discharge hospitals were considered as clusters.

**Table 1:** ICD9CM diagnosis and procedures description (and codes) for comorbidities.

Heart diseases
Rheumatic fever with heart involvement (391); Chronic rheumatic pericarditis (393); Diseases of mitral valve (394); Diseases of aortic valve (395); Diseases of mitral and aortic valves (396); Other rheumatic heart disease (398); Ischemic heart disease (410–414); Acute pericarditis (420); Acute and subacute endocarditis (421); Acute myocarditis (422); Other diseases of pericardium (423); Other diseases of endocardium (424); Cardiomyopathy (425); Cardiac dysrhythmias (427); Heart failure (428); Ill-defined descriptions and complications of heart disease (429); Bulbus cordis anomalies and anomalies of cardiac septal closure (745)
Syphilitic endocarditis (093.2); Diseases of tricuspid valve (397.0); Rheumatic diseases of pulmonary valve (397.1); Rheumatic diseases of endocardium; valve unspecified (397.9); Chronic pulmonary heart disease; unspecified (416.9); Atrioventricular block; complete (426.0); Anomalous atrioventricular excitation (426.7); Conduction disorder; unspecified (426.9); Certain sequelae of myocardial infarction; not elsewhere classified (429.7); Congenital stenosis of aortic valve (746.3); Congenital insufficiency of aortic valve (746.4); Congenital mitral stenosis (746.5); Congenital mitral insufficiency (746.6); Tachycardia; unspecified (785.0)
Atrioventricular block, unspecified (426.10); Mobitz (type) II atrioventricular block (426.12); Other second degree atrioventricular block (426.13); Mechanical complication of cardiac device, implant, and graft: Due to cardiac pacemaker (electrode) (996.01), Due to automatic implantable cardiac defibrillator (996.04)
Other personal history presenting hazards to health: Surgery to heart and great vessels (V15.1); Organ or tissue replaced by transplant: Heart valve (V42.2); Organ or tissue replaced by other means: Heart (V43.2), Heart valve (V43.3); Other postprocedural states: Cardiac device in situ (V45.0), Aortocoronary bypass status (V45.81), Percutaneous transluminal coronary angioplasty status (V45.82); Fitting and adjustment of other device: Cardiac device Reprogramming (V53.3)
*Procedure*
Operations on valves and septa of heart (35)
Removal of coronary artery obstruction and insertion of stent(s) (36.0); Bypass anastomosis for heart revascularization (36.1); Bypass anastomosis for heart revascularization (37.0); Cardiotomy and pericardiotomy (37.1); Pericardiectomy and excision of lesion of heart (37.3); Repair of heart and pericardium (37.4); Heart replacement procedures (37.5); Implantation of heart and circulatory assist system(s) (37.6); Other operations on heart and pericardium (37.9)
Cerebrovascular diseases
Cerebrovascular disease (430–438); Diseases of arteries, arterioles, and capillaries (440–448); Vascular insufficiency of intestine (557)
Aneurysm of aorta, specified as syphilitic (093.0)
Liver/chronic digestive disease
Regional enteritis (555); Ulcerative colitis (556); Acute and subacute necrosis of liver (570); Chronic liver disease and cirrhosis (571); Liver abscess and sequelae of chronic liver disease (572); Other disorders of liver (573); Viral hepatitis (070)
Esophageal varices with bleeding (456.0); Esophageal varices without mention of bleeding (456.1); Esophageal varices in diseases classified elsewhere (456.2); Diseases of pancreas (577.0–577.9)
Organ or tissue replaced by transplant: Liver (V42.7)
Chronic renal diseases
Chronic glomerulonephritis (582); Nephritis and nephropathy, not specified as acute or chronic (583); Acute kidney failure (584); Chronic kidney disease (CKD) (585); Renal failure, unspecified (586); Renal sclerosis, unspecified (587); Disorders resulting from impaired renal function (588)
Encounter for dialysis and dialysis catheter care (V56); Organ or tissue replaced by transplant: Kidney (V42.0); Other postprocedural states: Renal dialysis status (V45.1)
*Procedure*
Transplant of kidney (556)
Venous catheterization for renal dialysis (389.5); Hemodialysis (399.5); Peritoneal dialysis (549.8)
Anemia and coagulation disorders
Iron deficiency anemias (280); Other deficiency anemias (281); Coagulation defects (286)
Anemia; unspecified (285.9); Qualitative platelet defects (287.1); Primary thrombocytopenia (287.3); Secondary thrombocytopenia (287.4); Thrombocytopenia, unspecified (287.5)
Cancer
Malignant neoplasms (140–208)
Personal history of malignant neoplasm (V10)
COPD
Chronic obstructive pulmonary disease and allied conditions (490–496)

We studied two outcomes: 1) access to PTCA within 2 days (PTCA2d) after hospital admission for incident STEMI, using the following ICD9CM codes as principal or secondary procedure: 00.66, 36.01, 36.02, 36.05, 36.06, 36.07. Whenever there was a second hospitalisation within 2 days, PTCA procedures in the discharge record following the index record were also considered; 2) 30-day mortality due to natural causes (ICD9: 001–799) occurred within 30 days after the date of admission.

Only for descriptive purposes, we also considered the outcome access to PTCA within 90 minutes (PTCA90m) after hospital admission for incident STEMI. However, we did not model this information because it was registered only for the last four years and information on its quality was not available for this study.

### Statistical analysis

We carried out descriptive statistics of variables and tested for differences of means (t test) or proportions (chi-squared test) over time. To study temporal trends of the association between education or gender and access to PTCA within 2 days or 30-day mortality, we used logistic regression.

We explored effect modification of the exposures considered and time through stratification and using appropriate interaction terms of dummy variables. In particular, for time we used a dummy variable of equal time periods (from 21 October 2001 to 20 April 2007 and form 21 April 2007 to 20 October 2012). We then estimated crude and adjusted odds ratios (ORs) including education, gender, age at event, birthplace, comorbidities, stratifying by time period or single year. To study temporal trends of access to PTCA and occurrence of 30-day mortality, we included the dummy variable of time in the models and compared associations in the more recent period (after) toward the less recent period (before).

We used the Likelihood Ratio Test (LRT) to assess if the interactions were statistically significant, and the effect of adding confounding variables in the models.

In a further analysis, we used hierarchical logistic regression [26] to account for the presence of clustering of patients within hospitals: we considered individual characteristics as level-1 fixed predictors, and hospitals as level-2 random intercept predictors. In order to increase the accuracy of standard errors, we grouped hospitals with less than 30 records, obtaining more than 30 level-2 clusters. We did not apply this kind of analysis to models stratified by year.

We considered two-sided p-values less than 0.05 as statistically significant. We used software SAS Enterprise guide to perform statistical analyses.

## Results

We analysed 14,013 patients with STEMI discharged from 87 hospitals in Lazio region during the study period. The average number of PTCA90m by hospital (available only in 2008–2012) was 57 (range 1–236), while it was 199 for PTCA2d (1–1046), and 18 for the number of deaths (1–166).

**Fig 2:**
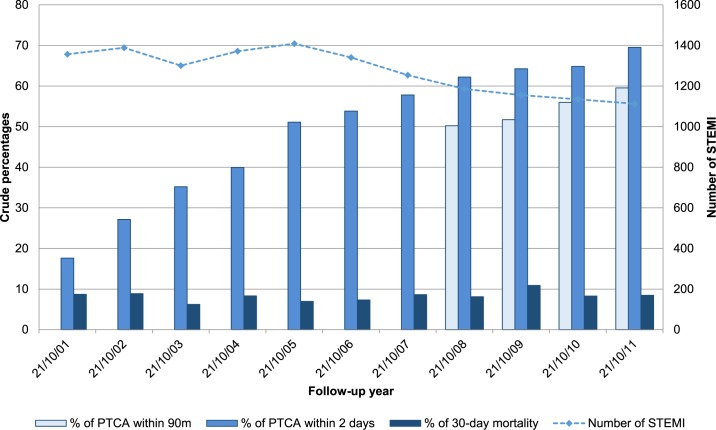
Number of STEMI, crude percentages of PTCA within 90 minutes and within 2 days, and percentage of 30-day mortality, by year of follow-up.

Fig 2 shows temporal trends of the number of STEMI, PTCA90m and PTCA2d crude percentages, and 30-day mortality.

Table 2 shows the patients’ characteristics and outcomes for each year considered. The number of STEMI decreased over time (from 1357 to 1113), while the percentages of PTCA procedures increased (from 50.2% to 59.5% for PTCA90m, p = 0.0015, and from 17.6% to 69.5% for PTCA2d, p<0.0001); 30-day mortality was 8.2% in the study period, with some variability over time (p = 0.0079). Mean age of the study population was 65 years, 72.9% were male, 50.7% were born in Rome, and the percentage of people with low education (no formal or only primary education) was 33.1%. Statistically significant differences of patients’ characteristics can be observed over time, except for birthplace. The prevalence of hospitalisation for comorbidities was 12.9% for heart, 7.0% for cerebrovascular diseases, 1.8% for liver/chronic digestive diseases, and 3.1% for chronic renal diseases, 1.5% for anemia and coagulation disorders, 4.9% for cancer, 3.8% for COPD. Percentages increased over time, except those for cerebrovascular and liver/chronic digestive causes, and for COPD, which showed a steady trend.

**Table 2:** Distribution of characteristics in patients with incident STEMI (mean and SD for age) by year at event.

	Total		Year*																						p**
			2001		2002		2003		2004		2005		2006		2007		2008		2009		2010		2011		
**Sociodemographic characteristics**	**n**	**%**	**n**	**%**	**n**	**%**	**n**	**%**	**n**	**%**	**n**	**%**	**n**	**%**	**n**	**%**	**n**	**%**	**n**	**%**	**n**	**%**	**n**	**%**	** **
Education																									
*None/Primary*	4636	33.1	466	34.3	499	35.9	482	37.0	440	32.1	474	33.6	439	32.7	435	34.7	376	31.7	362	31.3	359	31.6	304	27.3	0.0034
*Lower secondary*	4178	29.8	428	31.5	380	27.4	368	28.3	433	31.6	426	30.2	388	28.9	349	27.8	360	30.4	343	29.7	347	30.6	356	32.0	
*Upper secondary*	3533	25.2	320	23.6	345	24.8	310	23.8	345	25.1	340	24.1	337	25.1	312	24.9	300	25.3	323	27.9	298	26.3	303	27.2	
*Post-secondary or more*	1666	11.9	143	10.5	165	11.9	141	10.8	154	11.2	169	12.0	177	13.2	158	12.6	150	12.6	128	11.1	131	11.5	150	13.5	
Males	10218	72.9	1042	76.8	1055	76.0	948	72.9	1019	74.3	1029	73.0	992	74.0	888	70.8	866	73.0	797	68.9	803	70.7	779	70.0	<0.0001
Age (*mean and SD*)	*64*.*6*	*10*.*1*	*61*.*5*	*9*.*1*	*62*.*6*	*9*.*4*	*63*.*3*	*9*.*3*	*63*.*1*	*9*.*9*	*64*.*5*	*9*.*7*	*64*.*7*	*9*.*9*	*65*.*8*	*9*.*9*	*65*.*6*	*10*.*5*	*66*.*4*	*10*.*5*	*67*.*1*	*10*.*5*	*67*.*4*	*10*.*7*	<0.0001
Born in Rome	7109	50.7	703	51.8	668	48.1	643	49.4	684	49.9	707	50.2	689	51.4	635	50.6	621	52.4	614	53.1	571	50.3	574	51.6	0.3982
**Comorbidities**																									
Heart diseases	1804	12.9	172	12.7	209	15.0	168	12.9	190	13.8	180	12.8	183	13.6	189	15.1	126	10.6	150	13.0	125	11.0	112	10.1	0.0079
Cerebrovascular diseases	986	7.0	96	7.1	93	6.7	92	7.1	89	6.5	103	7.3	97	7.2	97	7.7	82	6.9	87	7.5	63	5.6	87	7.8	0.4481
Liver/chronic digestive diseases	251	1.8	26	1.9	31	2.2	23	1.8	20	1.5	24	1.7	24	1.8	23	1.8	23	1.9	21	1.8	15	1.3	21	1.9	0.8831
Chronic renal diseases	428	3.1	22	1.6	34	2.4	28	2.2	39	2.8	51	3.6	53	4.0	51	4.1	32	2.7	40	3.5	44	3.9	34	3.1	0.0021
Anemia and coagulation disorders	217	1.5	14	1.0	12	0.9	18	1.4	18	1.3	26	1.8	20	1.5	19	1.5	17	1.4	29	2.5	21	1.9	23	2.1	0.023
Cancer	681	4.9	43	3.2	62	4.5	52	4.0	78	5.7	79	5.6	65	4.8	62	4.9	64	5.4	61	5.3	57	5.0	58	5.2	0.0183
COPD	526	3.8	55	4.1	63	4.5	53	4.1	48	3.5	59	4.2	50	3.7	44	3.5	38	3.2	47	4.1	33	2.9	36	3.2	0.5966
**Outcomes**																									
PTCA within 90m^***^	1535	54.5	-		-		-		-		-		-		-		339	50.2	374	51.7	379	56.0	443	59.5	0.0015
PTCA within 2 days	6780	48.4	239	17.6	377	27.1	458	35.2	548	39.9	720	51.1	722	53.8	725	57.8	738	62.2	743	64.3	736	64.8	774	69.5	<0.0001
30-day mortality	1150	8.2	118	8.7	123	8.9	81	6.2	114	8.3	98	7.0	98	7.3	108	8.6	96	8.1	126	10.9	94	8.3	94	8.4	0.0079
**TOTAL**	**14013**	** **	**1357**	** **	**1389**	** **	**1301**	** **	**1372**	** **	**1409**	** **	**1341**	** **	**1254**	** **	**1186**	** **	**1156**	** **	**1135**	** **	**1113**	** **	** **

* Each year covers time span from 21 October to 20 November.

** P-values based on t test for age and chi-square for the other variables in association with time.

*** Data available only in 2008–2012, total observations = 2819: 675 in 2008, 723 in 2009, 677 in 2010, and 744 in 2011.

### Results from regression models

Crude ORs of PTCA2d were positively associated with either education or gender, resulting respectively 1.50 (95%IC 1.337–1.674) among the more educated compared to the less educated, and 1.53 (1.417–1.647) among males compared to females. On the contrary, ORs of mortality were inversely associated with either education or gender, resulting 0.54 (95%IC 0.433–0.665) (more educated vs less educated) and 0.63 (0.556–0.716) (males vs females).

#### Adjusted analysis and temporal trends of differentials

We did not find relevant interactions between the exposures considered, while when we analysed time we found interactions in association with PTCA2d for education (LRT = 0.022) and for gender (LRT = 0.066). Therefore, we calculated mutually adjusted ORs for demographic risk factors and concomitant diseases stratified by time periods (Table 3).

**Table 3:** Association between educational level or gender (crude, adjusted, and stratified OR and 95% CI) and the 2 outcomes studied. Years 2001–2012.

Risk factor	Time period 1		Time period 2		Crude analysis stratified by time period*								Adjusted** analysis stratified by time period*							
					Time period 1				Time period 2				Time period 1				Time period 2			
	Number of STEMI (7566)	Outcome %	Number of STEMI (6457)	Outcome %	OR	95% CI			OR	95% CI			OR	95% CI			OR	95% CI		
Outcome 1: PTCA within 2 days																				
Education																				
*None/Primary (ref*.*)*	2595	30.5	2041	55.9	1.00				1.00				1.00				1.00			
*Lower secondary*	2248	38.1	1930	65.7	1.40	1.245	-	1.580	1.51	1.329	-	1.718	1.14	1.006	-	1.294	1.03	0.898	-	1.294
*Upper secondary*	1847	39.6	1686	67.0	1.49	1.316	-	1.690	1.60	1.402	-	1.833	1.16	1.018	-	1.330	0.99	0.856	-	1.330
*Post-secondary or more*	866	42.0	800	62.1	1.65	1.408	-	1.935	1.29	1.094	-	1.530	1.36	1.157	-	1.607	0.87	0.724	-	1.607
Gender																				
*Females (ref*.*)*	1919	29.7	1876	52.0	1.00				1.00				1.00				1.00			
*Males*	5637	38.6	4581	66.8	1.49	1.329	-	1.661	1.86	1.663	-	2.07	1.30	1.157	-	1.461	1.57	1.395	-	1.765
Outcome 2: 30-day mortality																				
Education																				
*None/Primary (ref*.*)*	2595	10.4	2041	12.9	1.00				1.00											
*Lower secondary*	2248	6.4	1930	7.4	0.58	0.472	-	0.719	0.54	0.431	-	0.662	0.88	0.707	-	1.106	0.81	0.645	-	1.015
*Upper secondary*	1847	5.6	1686	7.0	0.51	0.400	-	0.641	0.51	0.404	-	0.636	0.84	0.651	-	1.070	0.88	0.690	-	1.121
*Post-secondary or more*	866	7.2	800	5.9	0.66	0.496	-	0.881	0.42	0.305	-	0.580	0.95	0.705	-	1.285	0.63	0.449	-	0.874
Gender																				
*Females (ref*.*)*	1919	10.3	1876	11.6	1.00				1.00				1.00				1.00			
*Males*	5637	6.8	4581	7.7	0.63	0.526	-	0.754	0.64	0.536	-	0.765	0.81	0.667	-	0.979	0.91	0.751	-	1.100

* Time period 1: from 21 October 2001 to 20 April 2007; time period 2: form 21 April 2007 to 20 October 2012.

** Mutually adjusted for education and gender, and adjusted for age at event, birthplace and comorbidities.

For PTCA2d, the stratified analysis showed educational differential in the first time period only (OR of higher vs lower education 1.36, 95%CI 1.157–1.607), adjusting for the other risk factors considered. We observed adjusted gender differentials in both time periods, with males’ ORs vs females’ ORs 1.30 (95%CI 1.157–1.461) in the first period and 1.57 (1.395–1.765) in the second one.

For 30-day mortality, we observed educational differentials as measured by point estimates in both time periods; however, such differences were statistically significant only in the second period and between the extreme categories, with the more educated advantaged compared to the less educated (OR 0.63, 95%CI 0.449–0.874). For the gender exposure we observed only borderline differentials in the first time period (OR 0.81, 95%CI 0.667–0.979).

**Fig 3:**
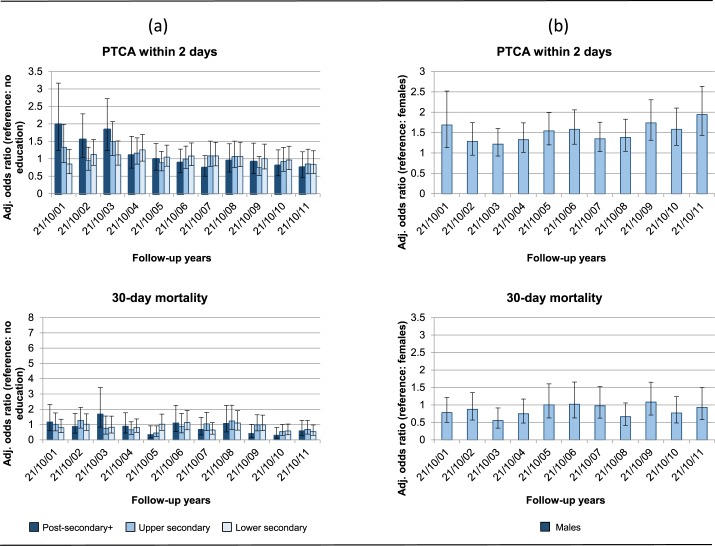
Association between educational level (a) or gender (b) (ORs mutually adjusted for education and gender, and adjusted for age at event, birthplace and comorbidities, and 95% CIs) and the 2 outcomes studied, by year. Years 2001–2012.

When we analysed adjusted ORs of educational level or gender on PTCA2d by year (Fig 3), we found inequalities for educational level at the beginning of the study period (in 2001–2003), and we observed higher ORs of PTCA2d for males compared to females in all years except in 2002 and 2003. Patterns of educational or gender differentials in 30-day mortality by year were rather stable and suggested an advantage of patients with high educational level and of males compared to their counterparts.

#### Adjusted temporal trends of access to PTCA and occurrence of 30-day mortality

The use of PTCA2d increased over the two time periods considered (after vs before), with adjusted ORs 3.45 (95%CI 3.203–3.713), while mortality did not show association with time (0.90, 95%CI 0.786–1.019).

When we applied the hierarchical logistic regression to take into account hospitals as clusters, we did not find relevant differences in comparison with previous findings from logistic regression.

## Discussion

Using data from a large census-based cohort of residents in Rome (Italy) over 11 years, we provide updated evidence on educational and gender differentials of access to PTCA and 30-day post-STEMI mortality, which was the central objective of our study. We used a valid exposure measure of individual educational level from census data.

Main results show that the use of PTCA among patients with a first occurrence of STEMI increased during the study period, while we did not find changes in 30-day mortality. We also observed a decreasing number of STEMI, which may be partially explained with the closed cohort design. This finding is however also in agreement with recent national [21] and international literature [5, 27, 28], which attributes a decrease in the number of STEMI to the improvement of primary prevention measures, and the increase of evidence-based therapeutic changes.

We observed a clear pattern of educational differentials in the access to PTCA therapy (lower among the less educated), but only in the first time period, accompanied by a less evident pattern of 30-day mortality (higher among the less educated).

Gender differences were also evident in both time periods for the access to PTCA (lower among women), while the pattern was less evident for 30-day mortality (higher among women).

Given the variability of the number of procedures delivered by discharge hospitals and their intrinsic characteristics, we repeated the analysis using a hierarchical approach to account for the hospital as a cluster, obtaining similar results.

The increased use of revascularization over time has been already documented in studies conducted in a large Italian region (Tuscany) in 2001–2008 [19] and in 1997–2010 [21]; the more recent study showed a steady short-term prognosis of STEMI when STEMI and NSTEMI were analysed separately. These results may appear contradictory, as one would expect decreasing mortality pattern following increased use of evidence-based cardiologic interventions. However, such results should be interpreted taking into account changes occurred throughout the study period in our region in factors related to the STEMI system of care indicated in recent guideline for the management of STEMI [24], affecting 30-day mortality in opposite directions: 1) the increased awareness of professionals involved in the health care and assistance of patients with cardiac problems, which may decrease the severity of acute events and therefore the mortality risk; 2) the improved management of acute coronary events implies changes in patients selection processes over time, which on the one hand may increase the survival of more severe STEMI and their chance to be treated before dying over time, but on the other hand may impact negatively on the overall short-term prognosis observed in our data [29, 30].

Our findings show differences in the treatment of patients with STEMI depending on educational background in Rome only in the first time period considered, in analogy with the results registered a decade ago [18], suggesting an improvement in the ability of the health system to reach the whole population.

Coronary risk factors, refusal to give consent for the intervention, misinterpretation of symptoms, and therefore delays in medical assistance, have been invoked as possible explanations of such differences [31, 32, 33]. The spread-out of revascularization procedures over time may have played a role in the reduction of such differentials. Also in the study conducted in Tuscany in 2001–2008, findings showed a reduction of the socioeconomic differential in the PTCA utilisation which were explained by diffusion of suitable invasive cardiology units and improved organizational models of care.

Equity of treatment is suggested also by lack of strong educational differentials in short-term mortality after STEMI, which is a robust indicator of appropriate hospital care. Educational differentials in mortality observed among our STEMI cohort only in the more recent times and between the extreme categories of educational level, may be partially explained with factors not included in our analysis, such as improved medical treatment and increased survival of high-risk patients in particular among patients with the highest education [29]. In addition, factors related to the increase of unemployment and impoverishment of the population after the economic downturn occurred in the more recent years, which may independently worsen the population health status, may contribute to explain this result [34, 35].

In part our results are also comparable with those from the study conducted in Piedmont [20] where, although socioeconomic differences in the use of revascularization were observed, no differences emerged for in-hospital and 1-year mortality, suggesting that patients were differently, but equitably, treated.

We observed differentials in treatment of STEMI by gender, with women being more disadvantaged than men. Gender disparity in revascularization not depending on severity of illness or risk of mortality has been previously observed in a large study conducted in Piedmont in 2009 [36] and in the more recent study conducted in Tuscany, and ascribed to atypical clinical symptoms, anatomical characteristics of vessels, and underestimation of cardiac ischemia among women [21]. Also the international literature reported gender differentials in revascularization in women [16, 14], which were explained by atypical presentation of symptoms or higher bleeding compared to men, or by delays in request for medical help. We also observed higher mortality among females in the first time period considered in our study, in analogy with a study conducted in Sweden, which analysed in-hospital mortality in STEMI patients [37], and in the Netherlands, which analysed short-term prognosis among AMI patients [9]. However, the evidence available on this gender issue is controversial. We did not observe differences in the second time period considered, consistently with the results of an Italian study in Piedmont which did not find gender differentials in 2008 [20]. A recent study using Cardiovascular Magnetic Resonance Imaging in STEMI patients reperfused by primary angioplasty found that unadjusted gender differences in early death after STEMI disappeared when differences in baseline risk and clinical characteristics were taken into account [38].

### Limitations

Sources of potential bias should be mentioned. Loss to follow-up due to emigration may introduce selection bias of the study population, in particular if emigrants differed significantly compared to non-emigrants in educational background or gender. Assuming that the more educated and males are more likely to emigrate, and that access to therapy and favourable prognosis is positively correlated with emigration risk, observed associations may be underestimated. However, in our cohort the probability of emigration by educational level or gender showed only modest heterogeneity: people who emigrated were 11.2% among those with the highest level of education vs 9.9% among those without formal education or owing the primary level, and were 12.8% among males vs 10.6% among females.

Another potential bias may arise from our definition of incident STEMI, as we checked for non-incident cases during a minimum period of five years before the cohort enrolment in 2001, implying that less recent STEMI had more chance to be misclassified as incident STEMI than more recent ones. However, a five-year period should be enough to find genuine incident cases over the study period.

Another limitation concerns the use of PTCA within 2 days after STEMI, which was the only valid information available for the whole study period. This information tells us that patients underwent PTCA within 2 days after an access to hospital for STEMI, and includes those patients who underwent PTCA within 90 minutes. Unfortunately, this measure may be considered only a proxy of timely access to PTCA. However, since it increased during the study period (from 17.6 to 69.5 out of hospitalized STEMI patients), and the PTCA within 90 minutes increased as well during the period we could measure it (between 50.2 to 59.5, during the last four years of observation), we think that the proxy measure is quite representative of an improvement of quality of care to patients with STEMI in our Region.

A source of misclassification may be related to the measurement of timely access to PTCA, which is known to be important in order to obtain the maximum benefit from the therapy [39]. We were able to measure only the time between hospital admission and PTCA, while the time elapsed between the arrival at the emergency room of the initial referral hospital and the intervention would be a more appropriate measure of timely access to PTCA. Under the hypothesis of non-differential misclassification then the observed differences would be underestimated.

In addition, we ignore some important confounding factors, such as the actual time elapsed between the onset of symptoms and the procedure, or pre-hospital delay, which may confound the associations observed [40]. For other important confounding risk factors, such as smoking and bad health status of the patients, we have considered the history of other hospitalisations (including BPCO) as proxies.

External validity of our results may be limited because of the closed cohort design approach, which however does not affect internal validity.

## Conclusions

We observed increased use of PTCA procedures and steady short-term prognosis after STEMI over an eleven-year follow-up. We observed educational differentials in PTCA only in the first time period, and women seemed disadvantaged, while we did not observe relevant educational or gender differentials in 30-day mortality, coherently with national and international literature. This study suggests that in the Lazio region there is equity of treatment in the access to PTCA, which indicates good quality of care in this area. Although gender differentials may be due to unaccounted clinical factors, it may be important to better assess the role of female gender in patients with STEMI both from a social and a clinical point of view.
